# Factors Associated with Sustained Exergaming: Longitudinal Investigation

**DOI:** 10.2196/13335

**Published:** 2019-07-31

**Authors:** Erin Kathleen O'Loughlin, Tracie A Barnett, Jennifer J McGrath, Mia Consalvo, Lisa Kakinami

**Affiliations:** 1 Concordia University Montreal, QC Canada; 2 Centre de Recherche du Centre Hospitalier de l'Université de Montréal Montreal, QC Canada; 3 Le Centre L'Institut National de la Recherche Scientifique–Institut Armand-Frappier Laval, QC Canada

**Keywords:** video games, physical activity, adolescents

## Abstract

**Background:**

Exergaming is technology-driven physical activity (PA) which, unlike traditional video game play, requires that participants be physically active to play the game. Exergaming may have potential to increase PA and decrease sedentary behavior in youth, but little is known about sustained exergaming.

**Objective:**

The objectives of this study were to describe the frequency, correlates, and predictors of sustained exergaming.

**Methods:**

Data were available in AdoQuest (2005-11), a longitudinal investigation of 1843 grade 5 students in Montréal, Canada. This analysis used data from grade 9 (2008-09) and 11 (2010-11). Participants at Time 1 (T1; mean age 14 years, SD 0.8 ) who reported past-week exergaming (n=186, 19.1% of AdoQuest sample) completed mailed self-report questionnaires at Time 2 (T2; mean age 16 years, SD 0.8). Independent sociodemographic, psychological, and behavioral correlates (from T2)/predictors (from T1 or earlier) were identified using multivariable logistic regression.

**Results:**

Of 186 exergamers at T1, 81 (44%) reported exergaming at T2. Being female and having higher introjected regulation (ie, a type of PA motivation indicative of internalizing PA as a behavior) were independent correlates. None of the predictors investigated were associated with sustained exergaming.

**Conclusions:**

Almost half of grade 9 exergamers sustained exergaming for 2 years. Exergaming may be a viable approach to help adolescents engage in and sustain PA during adolescence. Sex and PA motivation may be important in the sustainability of exergaming.

## Introduction

### Background

Only 8% of Canadian youth meet current physical activity (PA) recommendations [1] and they spend up to 9 hours sitting daily, often in screen-time pursuits [1,2]. Youth who do not meet PA recommendations are unlikely to benefit from the positive effects of PA on health [3,4], and sedentary youth may experience a wide range of negative physical and mental health issues [5]. Effective programs and public health policy are needed to address these issues because co-occurrence of low PA and high sedentary time contributes to childhood obesity and its numerous, deleterious health sequelae [6-9]. Despite possible negative impacts on health, screens are an indisputable reality in today’s increasingly high-tech world. Underscoring its popularity, 83% of US youth aged 8 to 18 years have a traditional video game console at home, and many of these consoles having add-on capacity for exergaming (ie, video games that are also a form of exercise) equipment. In addition, approximately 50% of adolescents own smartphones, which can download apps for mobile exergaming [10]. Many smartphone apps are free or low-cost, making exergaming and augmented reality (ie, a real-world view of the environment which is “augmented” by computer-generated input) highly accessible to youth.

In 2010, nearly 40% of US students in grades 9 to 12 exergamed at least 1 day per week [10], 25% of Canadian youth aged 15 to 18 years reported exergaming in the past week [11], and up to 20% of young adults exergamed 1 to 3 times per month or more [12]. In addition, in 2014, a representative sample of 20,122 Canadian youth in grades 6 to 10 reported exergaming for an average of 30 min per day [13]. The Xbox 360’s Kinect unit (ie, a motion-sensing device used to capture bodily movement and promote PA) sold approximately 25 million units in 2013 [14], and Pokémon Go, released on July 6, 2016 by Nintendo, had 25 million active players daily after only 1 month of the official release [15]. Leveraging benefit from screen fervor by incorporating PA into video gaming and designing exergames [16-19] that are easily available and appealing holds promise in terms of increasing PA and reducing sedentary time [16-19]. Exergaming may be a healthier pastime than traditional video games because they elicit more energy expenditure (EE) [20-22], and they may improve physical fitness, body composition, and cognitive health [20,23-25]. The effect of exergaming on reducing sedentary behavior has also been studied, but to a much lesser extent [26]. Overall, the data suggest that exergaming may be a viable method to increase PA, change body composition, and improve mental health, and it represents an improvement in EE over sedentary behavior [16,18,19,27].

There are important methodological challenges in the current literature evaluating exergaming including that many intervention studies report high attrition. Short intervention periods (ie, usually 6 or 12 weeks with no follow-up post intervention) may hinder detection of longer-term effects [24,28,29], and most studies are performed under controlled conditions that may not apply to “real-life” settings. For example, a small pilot study that provided African-American and Hispanic children with access to exergaming at home and during an after-school program indicated that fitness improved after 12 weeks [30]. However, 42% of participants lost interest in exergaming within 3 months, indicative that use related partly to the novelty of the consoles. This suggests that improving understanding of PA motivation pertaining to exergaming and sustained exergaming is needed.

### Objectives

To inform the development of exergaming interventions that have the potential to be sustained, evidence is needed on the determinants of sustained exergaming in naturalistic settings. Although the study by O’Loughlin et al [12]. reported that exergamers were more likely to be girls, to play nonactive video games, to watch TV 2 hours per day or more, to report weight-related stress, and to be nonsmokers, no reports to date describe factors associated with sustained exergaming in “real-world” contexts. The objectives of this study were (1) to describe the sustainability of exergaming over 2 years in a population-based sample of adolescents and (2) to identify factors associated with sustained exergaming.

## Methods

### Population Sample

Data were drawn from AdoQuest I (2005-11), a 6-year longitudinal study of grade 5 students (n=1843 aged 10-11 years at baseline), which investigated the natural course of the co-occurrence of health-compromising behaviors such as smoking, PA, sedentary behavior, and substance use in children [11]. A random sample of French-language elementary schools with more than 90 students in grade 5 was recruited in the greater Montreal area. To balance representation of students in high, middle, and low socioeconomic status, all schools were first stratified into groupings defined by tertile cut-offs of a school deprivation indicator based on maternal education, parental employment, and a measure of low family income that accounts for family size and area of residence [30]. An equal number of schools was then selected into each grouping, and 10 schools in the first, 10 in the second, and 9 in the third grouping (72.5% (29/40) of schools invited) agreed to participate. All students in all grade 5 classes in the 29 participating schools were eligible for recruitment. Of 2946 grade 5 students, 61.13% (1801/2946) participated at baseline (42 students joined after baseline data were collected). No data were collected from students who did not participate. Characteristics of the AdoQuest sample were comparable to those of 2 provincially representative samples of similarly aged Québec youth [31]. Parents completed mailed self-report questionnaires in 2006-07 and again in 2008-09. Participants provided assent, and their parents or guardians provided informed consent. The study received approval from the ethics and protection review boards of Concordia University and the Centre de Recherche du Centre Hospitalier de l’Université de Montréal.

Sustainability of exergaming over 2 years was investigated using data collected in the fall and winter of 2008-09 (Time 1 [T1]) when most participants were in grades 8 and 9 (aged 12-14 years) and 2 years later in the fall and winter of 2010-11 (Time 2 [T2]) when most participants were in grades 10 and 11 (aged 14-19 years). Data on exergaming and potential correlates and predictors were collected in mailed self-report questionnaires at both T1 and T2. Most questions used in AdoQuest were drawn from ongoing surveys and studies of youth including the Canadian Youth Smoking Survey [32] and the Nicotine Dependence in Teens study [33].

### Study Variables

#### Sustained Exergaming

At T1, participants were asked: “How many hours a day do you play active video games?” Response choices included 0<1, ≥1-2, ≥2-<3, ≥3-4, or ≥5 hours per day. Participants were categorized as exergamers if they responded ≥1 hour per day. At T2, participants were asked: “Do you play active video games (eg, Wii Fit, Dance Dance, and Revolution)?” (yes, no). Participants were categorized into 1 of 2 groups based on their responses to these 2 questions. “Sustained exergamers” included participants who reported exergaming at T1 and T2; those who “stopped exergaming” reported exergaming at T1 but not T2. “Never exergamers” and those who began exergaming between T1 and T2 were not included in the analysis.

#### Frequency, Timing, and Intensity of Exergaming

At T2, questions on exergaming were included based on the International Physical Activity Questionnaire (IPAQ), a short, self-administered questionnaire used in cross-national monitoring of usual weekly PA in youth and adults. The IPAQ demonstrates reliability as well as validity against accelerometer [34]. Specifically, exergamers were asked the following questions:

1. How many days a week do you play active video games? *(1-7 days)*
2. How many minutes (on average) do you play each time? *(open-ended)*
3. Physical effort during play? *(light, moderate, vigorous)*

Potential correlates and predictors of sustained exergaming were selected based on factors known to be associated with PA or exergaming in adolescence [12,35] and on the availability of data in AdoQuest.

Potential predictors measured before or at T1 included lifestyle behaviors (ie, level of PA, ever smoked cigarettes, binge drank, marijuana use, hours of TV per day, hours of computer per day, hours of nonactive video games per day, hours of sedentary behavior per day), weight-related indicators (ie, body mass index [BMI], stress about weight, perceived weight too heavy, trying to lose weight, body-related guilt, body-related shame), depressive symptoms, and the Pediatric Daytime Sleepiness Scale (PDSS) [36].

Potential correlates measured at T2 included sociodemographic characteristics (ie, age, sex, mother university-educated, annual household income, participant currently employed), lifestyle behaviors (ie, moderate-to-vigorous PA [MVPA] per week, meeting MVPA guidelines, past-year binge drinking, past-year marijuana use, hours of TV per day, hours of computer per day, hours of nonactive video games per day), weight-related indicators (BMI, stress about weight, perceived weight too heavy, trying to lose weight, body-related guilt, body-related shame), PA motivation, and depressive symptoms. The only variable reported by parents was mother’s education; all other variables were reported by participants. Multimedia Appendix 1 describes each variable in detail including response options, coding for analysis, and Cronbach alpha for scales.

### Data Analysis

Descriptive statistics were used to compare participants who did and did not sustain exergaming. The analysis considered investigation of each potential correlate or predictor as an independent study that addressed a specific hypothesis so that only 3 statistical tests (ie, univariate, partially adjusted model, and fully adjusted model) were performed for each potential correlate or predictor [34]. Potential confounders were retained in the model if they were not on the causal pathway and if they were correlated with the potential correlate or predictor and outcome at *r*>0.20 [35,37]. A total of 3 logistic regression models including (1) a univariate model examining the unadjusted association between the potential correlate/predictor and outcome with no covariates; (2) a partially adjusted model accounting for age and sex; and (3) a fully adjusted model including age, sex, and (other) potential confounders. Data were analyzed using SPSS version 20.0 (released 2011, SPSS Statistics for Windows; IBM Corp). All statistical tests were 2-sided, with the significance level set at 0.05.

## Results

Data on exergaming were collected in AdoQuest for the first time in grade 9 in 2008-09. Of 1801 grade 5 participants at inception in 2005-06, 68.46% (1233/1801) completed questionnaires at T1 in grade 9, and 16.46% (203/1233) of participants reported exergaming. At T2 in grade 11, 69.01% (1243/1801) completed questionnaires, and 23.97% (298/1243) of participants reported exergaming. A total of 54.08% (974/1801) participants completed questionnaires at both T1 and T2. Of the 974, 62.92% (613/974) never exergamed, 17.96% (175/974) began exergaming between T1 and T2, and 8.00% (81/974) and 10.98% (105/974) sustained and stopped exergaming, respectively. The total number of exergamers at T1 who provided data on exergaming at T2 was 19.09% (186/974), and these participants constituted the analytic sample. Table 1 compares the characteristics of the 186 participants retained in the analytic sample with those of the 59 participants who exergamed at T1 but were missing data on exergaming at T2. There were no statistically significant differences between the 2 groups, although 49.46% (92/186) of those retained reported taking action to change their weight compared with 36.02% (21/59) of those not retained (*P*=.08).

**Table 1:** Comparison of baseline characteristics of exergamers retained (n=186) and not retained (n=59) in the analytic sample, AdoQuest 2005-12.

Variable	Retained	Not retained^a^	*P* value
Age (years) at baseline, mean (SD)	10.8 (0.5)	10.7 (0.5)	.52
Male, n (%)	90 (48.6)	32 (53.4)	.52
Mother university-educated, n (%)	56 (30.1)	15 (25.5)	.13
French-speaking, n (%)	165 (88.6)	51 (86.4)	.66
Self-esteem, mean (SD)	2.0 (0.7)	2.0 (0.7)	>.99
Depressive symptoms, mean (SD)	2.0 (0.7)	2.0 (0.6)	.87
School connectedness, mean (SD)	2.3 (1.0)	2.3 (0.9)	.97
Taking action to change weight, n (%)	92 (49.4)	21 (35.8)	.08
TV ≥3 hours/day, n (%)	70 (37.6)	23 (39.7)	.78
Perceived academic performance, n (%) above average	72 (38.8)	27 (45.5)	.35

^a^Exergamers at T1 without exergaming data at T2.

The mean age of participants in the analytic sample was 16.7 (SD 0.5) years at T2, 42% (522/1243) were boys, 93% (1156/1243) were white, 76% (945/1243) were in grade 11, 47% (584/1243) were employed full or part-time, and 33% (410/1243) had university-educated mothers. The mean (SD) BMI was 23.6 (5.0) in boys and 22.2 (4.1) in girls. A total of 30% (373/1243) of participants’ parents reported an annual household income greater or equal to Can $100,000.

Of the 186 participants retained in the analytic sample, 43.6% (81/186) sustained exergaming (ie, reported exergaming at T1 and T2). At T2, sustained exergamers (n=81) exergamed on a mean (SD) of 1.9 (1.4) days per week, for 49.0 (33.8) min per bout on average. Overall, 35% (65/186) of sustained exergamers reported that they exergamed at light intensity, 42% (78/186) exergamed at moderate intensity, and 23% (32/186) exergamed at vigorous intensity.

In univariate analyses of potential correlates measured at T2 (Table 2), girls were twice as likely to sustain exergaming as boys. With each unit increase in depressive symptoms, the odds of sustained exergaming increased by 40%. Similarly, the odds of sustained exergaming were higher in participants with higher levels of introjected, identified, or intrinsic regulation. Finally, the odds of exergaming increased by 30% with each unit increase in body-related guilt. In partially adjusted models, girls were more likely to sustain exergaming, and for each unit increase in introjected regulation, there was a 60% increase in the odds of sustained exergaming. In fully adjusted models, the only variable retained as statistically significant was introjected regulation (odds ratio [OR] 1.8; 95% CI 1.1-3.2).

**Table 2:** Odds ratio (OR) and 95% CI for the association between potential correlates and sustained exergaming, AdoQuest 2005-12 (N=186).

Indicators^a^			n (%^b^)	Sustained exergaming^c^, n (%)	OR_crude_ (95% CI)	Model adjusted for age and sex OR_adj_ (95% CI)	Fully adjusted model OR_adj_ (95% CI)	Covariate(s) included in fully adjusted model
**Sociodemographic indicators**								
	**Age (years)^d^**							
		14.39-16.63	60 (32.3)	25 (41.7)	1.3 (0.8-2.1)	1.2 (0.7-2.0)	1.2 (0.7-2.0)	Sex
		16.64-16.98	59 (31.7)	21 (40.7)	—^e^	—	—	—
		16.99-19.86	59 (31.7)	28 (47.5)	—	—	—	—
	**Sex**							
		Boys	83 (44.6)	30 (34.9)	reference	reference	reference	Age, MVPA^f^, nonactive video games
		Girls	103 (55.4)	51 (50.5)	2.0 (1.1-3.4)	1.9 (1.0-3.4)	1.6 (0.8-3.3)	—
	**Mother university-educated**							
		Yes	49 (26.3)	23 (46.9)	reference	reference	reference	Age, sex, income
		No	108 (58.1)	45 (41.7)	0.8 (0.4-1.6)	0.8 (0.4-1.)	0.9 (0.4-2.0)	—
	**Income, Can $**							
		<100K	84 (45.2)	33 (38.9)	reference	reference	reference	Age, sex, mother’s education
		≥100K	60 (32.3)	28 (46.9)	1.4 (0.7-3.0)	1.4 (0.7-3.0)	1.5 (0.7-3.2)	Age, sex, mother’s education
	**Employed (AdoQuest participant)**							
		Yes	78 (42.0)	34 (43.6)	reference	reference	reference	Age, sex
		No	101 (54.3)	43 (42.6)	1.0 (0.5-1.7)	1.0 (0.6-1.9)	1.0 (0.6-1.9)	Age, sex
**Lifestyle behaviors**								
	**MVPA/min per week^d^**							
		0-105	59 (32.8)	21 (35.6)	1.0 (1.0-1.0)	1.0 (1.0-1.0)	1.0 (1.0-1.0)	Age, sex, identified regulation
		106-295	57 (30.6)	30 (52.6)	—	—	—	—
		≥295	64 (34.4)	28 (43.8)	—	—	—	—
	**Meets MVPA guidelines**							
		Yes	42 (22.6)	16 (38.1)	reference	reference	reference	Age, sex, intrinsic, body shame
		No	138 (74.2)	63 (45.7)	1.4 (0.7-2.8)	1.1 (0.5-2.3)	1.3 (1.0-1.6)	—
	**Binge drank past year**							
		Yes	82 (44.1)	37 (45.1)	reference	reference	reference	Age, sex, marijuana use, intrinsic regulation
		No	94 (50.5)	39 (41.5)	0.9 (0.5-1.6)	0.9 (0.5-1.6)	1.2 (0.6-2.4)	—
	**Used marijuana past year**							
		Yes	141 (75.8)	71 (50.0)	reference	reference	reference	Age, sex, binge drinking, smoked cigarettes
		No	34 (18.3)	14.2 (41.8)	1.0 (0.8-1.3)	0.7 (0.3-1.6)	0.4 (0.1-1.0)	—
	**Hours of TV/day^d^**							
		≥0<1	42 (22.6)	19 (45.2)	1.0 (0.8-1.4)	1.1 (0.8-1.4)	1.1 (0.8-1.5)	Age, sex, depressive symptoms
		≥1<2	78 (41.9)	31 (39.7)	—	—	—	—
		≥2	66 (35.5)	31 (47.0)	—	—	—	—
	**Hours of computer/day^d^**							
		≥0<1	38 (20.4)	15 (39.5)	1.1 (0.9-1.3)	1.1 (0.8-1.3)	1.1 (0.8-1.3)^g^	Age, sex
		≥1<2	56 (30.1)	22 (39.3)	—	—	—	—
		≥2	92 (49.5)	44 (47.8)	—	—	—	—
	**Hours of nonactive video games/day^d^**							
		0	82 (44.1)	32 (39.0)	1.0 (0.8-1.3)	1.0 (0.9-1.4)	1.0 (0.9-1.4)^g^	Age, sex
		<1	50 (26.7)	28 (56.0)	—	—	—	—
		≥1	53 (28.5)	21 (39.6)	—	—	—	—
**Weight-related indicators**								
	**BMI^d,h^**							
		16.01-20.1	49 (26.3)	23 (46.9)	1.0 (0.9-1.0)	0.9 (0.9-1.1)	0.9 (0.8-1.0)	Age, sex, income, extrinsic motivation, body shame, body guilt
		20.2-23.54	51 (27.4)	20 (39.2)	—	—	—	—
		23.55-40.70	49 (26.3)	18 (36.7)	—	—	—	—
	**Stressed about weight**							
		Yes	50 (26.9)	25 (50.0)	reference	reference	reference	Age, sex, depressive symptoms, weight perception, introjected regulation, external regulation, body shame, body guilt, trying to lose weight
		No	129 (69.4)	52 (40.3)	0.7 (0.4-1.3)	0.8 (0.3-2.1)	0.9 (0.3-2.0)	—
	**Perceived weight status: overweight**							
		Yes	49 (26.3)	23 (46.9)	reference	reference	reference	Age, sex, BMI, introjected regulation, external regulation, body shame, body guilt, stress about weight, trying to lose weight
		No	127 (68.3)	53 (41.7)	0.8 (0.2-1.6)	0.8 (0.2-1.6)	0.3 (0.1-1.3)	—
	**Trying to lose weight**							
		Yes	114 (61.3)	57 (50.0)	reference	reference	reference	Age, sex, BMI, weight perception, introjected regulation, external regulation, identified regulation, stress about weight, weight perception, body shame, body guilt
		No	62 (33.3)	25 (39.5)	0.7 (0.4-1.2)	0.8 (0.4-1.4)	0.8 (0.3-1.2)	—
	**Body-related guilt^d^**							
		0≤1.0	67 (36.0)	20 (29.9)	1.3 (1.0-1.7)	1.2 (0.9-1.7)	0.9 (0.4-2.0)	Age, sex, BMI, depressive symptoms, weight perception, extrinsic regulation, introjected regulation, identified regulation, body shame, stress about weight, trying to lose weight
		>1.0≤2.0	52 (28.0)	26 (50.0)	—	—	—	—
		>2.0-5.0	58 (31.2)	29 (50.0)	—	—	—	—
	**Body-related shame^d^**							
		0≤1.0	61 (32.8)	16 (26.2)	1.2 (0.9-1.7)	1.2 (0.9-1.6)	1.0 (0.4-2.3)	Age, sex, stress about weight, BMI, depressive symptoms, weight perception, extrinsic regulation, introjected regulation, body guilt, trying to lose weight
		1.0≤2.0	59 (31.7)	31 (52.5)	—	—	—	—
		>2.0-4.8	57 (30.6)	28 (49.1)	—	—	—	—
**Physical activity motivation**								
	**Amotivation^d^**							
		0≤1	130 (69.9)	57 (43.8)	1.3 (0.7-2.4)	1.3 (0.7-2.6)	2.0 (0.9-5.0)	Age, sex, identified regulation, intrinsic regulation, body shame
		>1-4	47 (25.3)	18 (38.3)	—	—	—	—
	**External^d^**							
		0≤1	113 (60.8)	46 (40.7)	1.0 (0.5-2.1)	1.1 (0.5-2.2)	0.9 (0.3-2.8)	Age, sex. BMI, depressive symptoms, stress about weight, weight perception, trying to lose weight, introjected regulation, body shame, body guilt
		>1≤1.67	20 (10.8)	11 (55)	—	—	—	—
		1.68-3.25	44 (23.7)	18 (40.9)	—	—	—	—
	**Introjected^d^**							
		0	67 (36.0)	21 (31.3)	1.7 (1.2-2.4)	1.6 (1.1-2.3)	1.8 (1.1-3.2)	Age, sex, depressive symptoms, weight stress, weight perception, external regulation, identified regulation, intrinsic regulation, trying to lose weight, body shame, body guilt
		1-1.67	46 (24.7)	20 (43.5)	—	—	—	—
		1.68-5	64 (34.4)	24 (53.1)	—	—	—	—
	**Identified^d^**							
		0-2.25	54 (29.0)	29 (37.0)	1.4 (1.0-1.8)	1.3 (1.0-1.8)	1.3 (0.8-2.0)	Age, sex, amotivation, introjected regulation, intrinsic motivation, regulation, body guilt, trying to lose
		2.26-3.25	63 (33.9)	22 (34.9)	—	—	—	—
		3.26-5	60 (32.3)	33 (55.0)	—	—	—	—
	**Intrinsic^d^**							
		0-1.0	56 (30.1)	19 (33.9)	1.3 (1.0-1.6)	1.3 (1.0-1.7)	1.3 (0.9-2.0)	Age, sex, MVPA, amotivation, identified, binge drinking, introjected regulation, body shame
		1-1.67	55 (29.6)	23 (41.8)	—	—	—	—
		≥1.68	66 (35.5)	33 (50.0)	—	—	—	—
**Depressive symptoms^d^**								
	0-1.67		59 (31.7)	22 (37.3)	1.4 (1.0-2.0)	1.3 (0.9-1.9)	1.4 (0.8-2.2)	Age, sex, introjected regulation, external regulation, body shame, body guilt, stress about weight, TV use
	1.68-2.50		66 (35.5)	25 (37.9)	—	—	—	—
	2.51-4.5		54 (29.0)	30 (55.6)	—	—	—	—

^a^Potential correlate measured in grade 11.

^b^Percentages may not total 100% due to missing data.

^c^Continuous variables were grouped according to tertile cut-offs for descriptive purposes.

^d^Potential correlate included in the model as a continuous variable. Odds ratio indicates the increase in the probability of the outcome per 1-unit change in the correlate.

^e^Not applicable.

^f^MVPA: moderate-to-vigorous physical activity.

^g^Partially and fully adjusted models were identical.

^h^BMI: body mass index.

In univariate analyses of potential predictors (Table 3), more time spent playing nonactive video games daily was protective against sustained exergaming sustainers. Trying to lose weight was associated with an 80% increase in the odds of sustained exergaming. In partially adjusted models controlling for sex and age and in fully-adjusted models, none of the potential predictors were statistically significantly associated with sustained exergaming.

**Table 3:** Odds ratio (OR) and 95% CI for the association between potential predictors and sustained exergaming, AdoQuest 2005-12 (N=186).

Predictors^a^			n (%^b^)	Sustained exergaming^c^, n (%)	OR_crude_ (95% CI)	Model adjusted for age and sex, OR_adj_ (95% CI)	Fully adjusted model, OR_adj_ (95% CI)	Covariates included in fully adjusted model
**Lifestyle behaviors**								
	**Physical activity level^d^**							
		1	37 (20.0)	13 (35.1)	1.0 (0.8-1.3)	1.1 (0.8-1.3)	1.1 (0.8-1.3)^e^	Age, sex
		2-3	107 (57.5)	50 (46.7)	—^f^	—	—	—
		4-5	40 (21.3)	16 (40.0)	—	—	—	—
	**Ever smoked cigarettes**							
		Yes	57 (30.7)	29 (50.9)	reference	reference	reference	Age, sex, cannabis, binge drinking, depressive symptoms, PDSS^g^
		No	127 (68.3)	51 (40.2)	0.7 (0.4-1.2)	0.7 (0.4-1.3)	0.5 (0.3-1.2)	—
	**Binge drank**							
		Yes	43 (26.3)	16 (37.2)	reference	reference	reference	Age, sex, cigarette, depressive symptoms, cannabis, PDSS
		No	143 (76.9)	65 (45.5)	1.4 (0.7-2.8)	1.3 (0.6-2.6)	1.5 (0.6-3.8)	—
	**Marijuana use**							
		Yes	21 (11.3)	9 (42.9)	reference	reference	reference	Age, sex, cig try, binge drinking, depressive symptoms
		No	164 (88.2)	72 (43.9)	1.0 (0.4-2.6)	0.9 (0.4- 2.4)	1.0 (0.3-3.5)	—
	**Hours of TV/day**							
		≤1	20 (10.8)	9 (45.0)	1.1 (0.6-2.2)	0.8 (0.6-1.1)	0.9 (0.6-1.4)	Age, sex, computer, sedentary behavior
		≥1≤3	93 (50.0)	41 (44.1)	—	—	—	—
		>3	72 (38.8)	30 (41.7)	—	—	—	—
	**Hours of computer/day^d^**							
		≤1	31 (16.7)	11 (35.5)	1.3 (1.0-1.7)	1.4 (1.0-2.0)	1.3 (0.9-2.1)	Age, sex, depressive symptoms, video games, TV, sedentary behavior
		≥1≤3	75 (40.3)	29 (38.7)	—	—	—	—
		>3	79 (42.5)	41 (51.9)	—	—	—	—
	**Hours of nonactive video games/day^d^**							
		≤1	107 (57.5)	54 (50.5)	0.6 (0.5-1.0)	0.8 (0.5-1.3)	0.8 (0.4-1.3)	Age, sex, computer, sedentary behavior
		>1	73 (39.2)	26 (35.6)	—	—	—	—
	**Hours of sedentary behavior/day^d^**							
		0-9.5	60 (32.3)	23 (38.3)	1.1 (1.0-1.2)	1.0 (1.0-1.1)	1.0 (0.9-1.2)	Age, sex, TV, computer, video games
		9.6-11.5	52 (27.4)	24 (46.2)	—	—	—	—
		>11.5	61 (37.8)	30 (49.2)	—	—	—	—
**Weight-related indicators**								
	**BMI^d,h^**							
		13.7-19.2	35 (18.8)	17 (48.6)	1.0 (0.9-1.1)	1.1 (1.0-1.2)	1.1 (1.0-1.2)	Age, sex, trying to lose weight, video games, perceived weight, stress about weight, sedentary behavior
		19.3-22.66	35 (18.8)	14 (40.0)	—	—	—	—
		22.67+	36 (19.4)	11 (30.6)	—	—	—	—
	**Stress about weight**							
		Yes	80 (43.0)	40 (50.0)	reference	reference	reference	Age, sex, depressive symptoms, trying to lose weight, perceived weight, PDSS, BMI
		No	105 (56.5)	41 (39.0)	0.6 (0.4-1.2)	0.8 (0.4-1.5)	0.6 (0.2-1.8)	—
	**Perceived weight too heavy**							
		Yes	55 (29.6)	16 (29.5)	reference	reference	reference	Age, sex, BMI, stress about weight, trying to lose weight
		No	128 (68.8)	39 (30.8)	1.1 (0.6-2.1)	1.1 (0.6-2.2)	3.0 (0.8-10.8)	—
	**Trying to lose weight**							
		No	113 (60.8)	42(37.2)	reference	reference	reference	Age, sex, stress about weight, depressive symptoms, perceived weight
		Yes	70 (37.6)	36 (51.4)	1.8 (1.0-3.3)	1.3 (0.7-2.6)	1.6 (0.7-3.5)	—
**Depressive symptoms^d^**								
	0-1.5		57 (30.6)	27 (47.4)	1.0 (0.7-1.4)	0.8 (0.5-1.2)	0.8 (0.5-1.3)	Age, sex, stress weight, binge drinking, trying to lose weight, PDSS, cigarettes, computer, sedentary behavior
	1.6-1.17		65 (35.0)	24 (36.9)	—	—	—	—
	1.8-5.0		62 (33.3)	30 (48.4)	—	—	—	—
**PDSS^d^**								
	0-7		62 (33.3)	24 (38.7)	1.0 (1.0-1.1)	1.0 (1.0-1.1)	1.0 (1.0-1.1)	Age, sex, stress about weight, binge drinking, depressive symptoms
	8-13		60 (32.3)	23 (38.3)	—	—	—	—
	≥14		62 (33.3)	34 (54.8)	—	—	—	—

^a^Potential predictor measured in grade 9.

^b^Percentages may not total 100% due to missing data.

^c^Continuous variables were grouped according to tertile cut-offs for descriptive purposes.

^d^Potential predictor included in the models as a continuous variable. Odds ratio (OR) indicates the increase in the probability of the outcome per 1 unit change in the predictor.

^e^Partially and fully adjusted models were identical.

^f^Not applicable.

^g^PDSS: Paediatric daytime sleepiness scale.

^h^BMI: body mass index.

## Discussion

### Principal Findings

The purpose of this study was to describe sustained exergaming in a population-based sample of adolescents and to identify factors associated with sustained exergaming. To our knowledge, this is the first investigation of sustained exergaming in a population-based sample. Results indicated that 44% of exergamers sustained exergaming for at least 2 years. Female sex and having higher introjected PA behavior regulation were associated with sustained exergaming.

In a cross-sectional study of 200 children who owned consoles, Simons et al [38] found that 11% never exergamed and only 32% exergamed regularly (ie, >1 hour per week). Because of concerns that the novelty of exergaming dissipates over time, there have been numerous calls to investigate the sustainability of exergaming [17,24,28,39-42]. The 44% (81/186) of sustained exergamers in our study compares with the 41% of girls (but not the 69% of boys) who remained involved in team sports for over 5 years, as observed by Belanger et al [41]. Specifically, of 1276 adolescents age 12-13 years who completed a 7-day PA recall every 3 months for 5 years, the authors reported that between 14% and 53% re-engaged in a specific PA after discontinuation. Although not investigated in the Belanger study, exergaming could be an intermittent activity in adolescents linked to the release of new games and consoles. Future studies will need more frequent follow-up to assess the stop-start aspect of exergaming over time.

Three-quarters (77% [143/186]) of participants exergamed at light or moderate intensity. Exergaming at these intensities may be more enjoyable, practical, and achievable among young persons. Thus, the flexibility of exergaming in level of intensity may contribute to sustainability.

Previous studies have reported that females are more likely to exergame than males [12], and in this analysis, girls were more likely to sustain exergaming. The reasons for sex differences in sustained exergaming may mirror the reasons for sex difference in exergaming. Girls, especially those with body image challenges, may be more comfortable being active at home away from the scrutiny of others, while still enjoying the social interaction provided by exergaming. Boys may be attracted by the novelty of exergaming but return to nonactive video games as the novelty wears off. Although sex differences in exergaming should be further investigated, our results suggest that exergaming may be a viable option to help girls in particular remain physically active.

A specific focus of this work was to assess whether PA motivation is associated with sustained exergaming. Self-determination theory (SDT) has been used as a framework to predict intentions to engage in and sustain traditional PA, and investigators have reported that those with higher intrinsic regulation (ie, motivation) report increased intentions to engage in PA than those with external PA regulation (eg, [43-46]. Introjected regulation (ie, a type of PA motivation indicative of internalization of PA as a behavior) was a correlate of sustained exergaming. Introjected regulation, as described in the SDT [47-49], is motivation from an internal pressure that usually drives short-term behavior change, but does not foster sustainable behavior change [48,50]. It is generally negatively associated with or unrelated to PA levels, although there appears to be sex differences such that girls who report higher introjected regulation also report more PA [49]. The link between behavior regulation and exergaming has been studied in clinical settings and in specific populations such as overweight youth [51,52], but few studies investigate this association in population-based samples of youth using the SDT [53], and it is not fully understood if exercise behavior regulation differs between those who do and do not sustain exergaming. Sustained exergamers may not be highly motivated intrinsically toward traditional PA and are drawn toward exergaming as a PA alternative. They may be interested in developing skills for fitness and weight change, which is fostered through fitness exergames (ie, body weight, body alignment, placement, feeling, and speed captured by motion and sensor-captures and displayed in real time on the screen) [52], or aspects of the advertising or marketing of fitness exergames may play on or increase guilt, particularly among female users. Future research should focus on better understanding the differences between motivation for PA and for exergaming, which in turn lead to exergaming interventions that have a greater impact on PA and sedentary behavior.

No predictors of sustained exergaming were identified in this study. Rather than being a planned behavior, exergaming may be triggered by events in the immediate present such as purchasing a new console or friends coming over, and therefore, has few predictors. It is also possible that the sample size was too small to detect factors associated with sustained exergaming because exergaming is a relatively new area of research, not all relevant predictors may not have been investigated.

Limitations of this study include the small sample size, that self-report data are subject to misclassification, that loss to follow-up may have resulted in selection bias, and that restriction of the sample to francophones may have limited external generalizability. As data were not collected more frequently, it was not possible to confirm whether participants exergamed between data collection cycles.

### Conclusions

Exergaming may represent a novel approach to help adolescents remain physically active during a period of life which is notable for sharp declines in PA. Exergaming may be more sustainable if games include components that foster intrinsic PA motivation such as providing more choices in the games offered (ie, with whom the game can be played, whether the setting is collaborative or competitive, how intensely the game is played) or having a coach and/or other social support while learning a new game. Finally, clinicians and practitioners can counsel parents to encourage their children to choose exergaming over more traditional sedentary video games or to exergame as a family.

## Appendix

Multimedia Appendix 1 Variables tested in models including response options, coding for analysis, and Cronbach alpha for scales.

## Abbreviations

BMI: body mass index
EE: energy expenditure
INSPQ: Institut national de santé publique du Québec
IPAQ: International Physical Activity Questionnaire
MVPA: moderate-to-vigorous physical activity
PA: physical activity
SDT: self-determination theory
