# Supplementary material for: Factors Associated with Sustained Exergaming: Longitudinal Investigation
Source: JMIR Serious Games. 2019 Jul 31;7(3):e13335. doi: 10.2196/13335 (PMC6786850; doi:10.2196/13335)
Supplement: Multimedia Appendix 1 [file games_v7i3e13335_app1.pdf]

Multimedia: Variables tested in models including response options, coding for analysis, and Cronbach's alpha for scales.			
	**Item(s)**	**Response choices/creation of score**	**Re-coded for analysis**
Age	Date of birth, Date of survey		
Sex	Are you a boy or a girl?	Male, Female	male, female
Smoke cigarettes	Have you ever in your life smoked a cigarette, even just a puff (drag, hit, haul)?	No, yes	No, yes
Currently employed	Are you currently working at a job or business (paid or unpaid)?	No, yes	No, yes
Binge drink	In the past 6 months, how often did you …? Drink 5 or more alcoholic beverages on one occasion	Never, just once to try, once a month, 2-3 times per month, 1times per week, 2-3 times per week, 4-6 times per week, every day	No, yes
Marijuana use	In the past 6 months, how often did you …? Use marijuana, cannabis, hashish	Never, just once to try, once a month, 2-3 times per month, 1times per week, 2-3 times per week, 4-6 times per week, every day	No, yes
Paediatric daytime sleepiness scale (PDSS)(1)<br><br>α =0.70	Usually, at what frequency do you…? (i)Fall asleep or feel drowsy while you're in class; (ii) Fall asleep or feel drowsy when you do your homework (iii) you're alert (awake) for the entire or almost the entire day (iv)You are tired and grumpy during most of the day (v) Do you have difficulties waking up in the morning? (vi) Do you fall back asleep when you are awake? (vii)Do you need someone to wake you up? (viii)Do you feel that you don't get enough sleep	1. Always<br>2. Often<br>3. Sometimes<br>4. Rarely<br>5. Never	Items were summed and divided by the number of items responded to create a score
Depressive symptoms (2-4)<br><br>α = 0.82	During the past 7 days how often have you: (i) felt too tired to do things; (ii) had trouble going to sleep or staying asleep; (iii) felt unhappy, sad or depressed; (iv) felt hopeless about the future; (v) felt nervous or tense; (vi) worried too much about things	1. Never 2. Rarely 3. Sometimes 4. Often 5. Always	Items were summed and divided by the number of items responded to create a score
Perceived weight status	Do you consider yourself……	1. Too thin 2. Normal 3. A bit too large 4. Much too large	No, yes
Stress about weight	In your lifetime, have you ever been stressed about your….weight	1. Never 2. A little 3. Some 4. A lot 5. Does not apply	No, yes
Trying to lose weight	What are you actually trying to do about your weight?	1. Lose weight; 2. Gain weight; 3. Not trying to change my weight	No, yes

BMI	Self-report height and weight	BMI = kg/m2 where kg is a person's weight in kilograms and m2 is their height in metres squared.	As is
Hours of TV daily	On average, about how many hours a day do you watch TV or videos?	1. <1; 2. 1-2; 3. 3-4; 4. 5-6; 5. ≥7	≥ 0 <1<br>≥1 <2<br>≥2
Hours of computer daily	How many hours do you usually use the computer games, or use the Internet in a single day?	1. <1; 2. 1-2; 3. 3-4; 4. 5-6; 5. ≥7	≥ 0<1<br>≥1<2<br>≥2
Hours of non-active videogames daily	How many hours do you usually play games in a single day?	1. <1; 2. 1-2; 3. 3-4; 4. 5-6; 5. ≥7	0<br><1<br>≥1
Physical activity weekly (5)	Which one of the following describes you the best for a normal week. Read all 5 statements before deciding on the one that best describes you.	1. All or most of my free time is spent doing things that involve little physical effort;<br>2. I sometimes (1-2 times per week) do physical activities in my free time; 3. I often (3-4 times per week) do physical activities in my free time,<br>4. I quite often (5-6 times per week) do physical activities in my free time; 5. I very often (7 or more times) do physical activities in my free time.	As is

1. Drake C, Nickel C, Burduvali E, Roth T, Jefferson C, Badia P. The pediatric daytime sleepiness scale (PDSS): sleep habits and school outcomes in middle-school children. Sleep. 2003 Jun 1;26(4):455-8.
2. Choi WS, Pierce JP, Gilpin EA, et al. Which adolescent experimenters progress to established smoking in the United States. Am J Prev Med 1997;13(5):385-391.
3. Escobedo LG, Reddy M, Giovino GA. The relationship between depressive symptoms and cigarette smoking in US adolescents. Addiction 1998;93(3):433-440.
4. Kandel DB, Davies M. Epidemiology of depressive mood in adolescents: an empirical study. Arch Gen Psychiatry 1982;39(10):1205–1212. doi:10.1001/archpsyc.1982.04290100065011
5. Kowalski KC, Crocker PR, Kowalski NP. Convergent validity of the physical activity questionnaire for adolescents. Pediatric exercise science. 1997 Nov;9(4):342-52.
